# Coping with the COVID-19 Crisis Through Faith-Based Strategies: A Multiple-Group Confirmatory Factor Analysis in Colombia

**DOI:** 10.1007/s10943-025-02538-6

**Published:** 2026-02-11

**Authors:** Diego Andrés Vásquez-Caballero, Leonardo H. Talero-Sarmiento, Ruth Natalia Suárez-Flórez

**Affiliations:** 1 Universidad Cooperativa de Colombia, Bucaramanga, Santander, Colombia; 2 Universidad Autónoma de Bucaramanga, Bucaramanga, Santander, Colombia; 3 Enlace Psicología, Bucaramanga, Santander, Colombia

**Keywords:** Religious coping, COVID-19, Coping strategies, Health crisis, Resilience

## Abstract

This study examines how adults in Colombia employed religious and other psychological coping strategies in response to the COVID-19 pandemic, with a specific focus on the role of faith-based practices in shaping adaptive responses. Drawing on Lazarus and Folkman’s transactional model of stress and coping and grounded in the Colombian context where religion holds strong cultural significance, we analyzed data from 5,559 participants using a psychometrically validated coping strategies scale with an approximate response rate around 62.1%. While the validation process ensured the reliability of the instrument, our focus was to explore how coping strategies clustered into distinct psychosocial profiles, how these profiles varied across sociodemographic subgroups, and how religious coping was integrated within each configuration. Cluster analysis identified four coping profiles, two of which featured strong religious engagement. Multiple-group confirmatory factor analysis confirmed measurement invariance across groups, supporting valid comparisons. The findings reveal that religious coping co-occurred to varying degrees with other strategies, such as problem-solving, emotional avoidance, and social or professional support, based on latent inter-factor correlations from an oblique confirmatory factor analysis. These results highlight the culturally embedded nature of faith in collective coping and contribute to the literature on religious resilience during large-scale public health crises.

**Supplementary Information:**

The online version contains supplementary material available at 10.1007/s10943-025-02538-6.

## Introduction

Coping strategies encompass diverse psychological and behavioral responses to stress that are shaped by sociocultural contexts. Among these, faith-based mechanisms occupy a central role in many communities, particularly during periods of acute collective adversity (Kroesbergen-Kamps, 2024). In Colombia, where religion has strong cultural prominence, studies by Plata et al. (2023) and Meza (2020) document both the intensification of individual faith practices and the rapid adaptation of communal celebrations during the COVID-19 crisis, underlining their importance for stress management. Convergent evidence indicates that religious practices can mitigate the effects of traumatic and challenging life events (Akanni et al., 2021; Kéri, 2024; Niu, 2024; Waila & Lindsay, 2024), and our data in Colombia suggest that faith-centered strategies were widely used and frequently combined with other responses to form complex coping patterns. Given the quantitative design, our analyses focus on concrete faith-based activities and routines rather than broader spiritual experiences, yet they remain informative for understanding how formal and informal religious practices contribute to well-being and everyday adaptation to adversity.

The COVID-19 pandemic generated unprecedented challenges for mental health worldwide and, in Colombia, profoundly affected economic, political, and social life (Hernández, 2020). This context heightened interest in social practices as coping mechanisms, with studies showing that faith-based activities not only mitigate psychological distress but also reinforce communal bonds and social cohesion, both crucial for coping with collective trauma (Asif et al., 2024). Conceptually, coping strategies are defined as mindsets and behaviors that enable individuals to navigate taxing circumstances (Cerquera et al., 2020). Lazarus and Folkman (1984) characterize coping as “ever-changing cognitive and behavioral efforts to manage specific demands assessed as taxing or exceeding one's resources” (p. 164), a framework that has anchored subsequent research showing that resilience depends on the effectiveness of these strategies and on cultural/contextual factors such as religion (Cerquera et al., 2020; González & Artuch, 2014). Conversely, inadequate coping responses, including avoidance and emotional dysregulation, have been linked to higher levels of depression and anxiety (Andreo et al., 2020; Lemos et al., 2019).

Within this broader architecture, religious practices provide potent tools for emotional regulation by offering comfort, resilience, and meaning-making. For instance, among older adults in Pakistan, higher engagement in faith-based activities reduced psychological distress and fear of COVID-19 (Asif et al., 2024), while research in Zambia highlights religious coping as a source of hope and perceived divine control amid pandemic uncertainty (Kroesbergen-Kamps, 2024).

The pandemic thus acted as a catalyst that intensified the need for adaptive coping strategies to address health risks, economic instability, and social isolation. Religious engagement emerged as a central resource alongside problem-focused and social coping, contributing to stress reduction and a sense of purpose (Alexandre et al., 2022; Caballero et al., 2021; Cheng & Chang, 2022; Couto et al., 2022). Religious practices afforded communal solidarity, existential reassurance, and perceived spiritual protection, particularly among university students and physicians, whose coping responses ranged from avoidance and isolation to active faith-based engagement and emotional sharing (Belchior et al., 2021; Portero de la Cruz et al., 2020).

These patterns are consistent with the findings of a recent meta-analysis by Pankowski and Wytrychiewicz-Pankowska (2023), which shows that positive religious coping was associated with better psychological outcomes during COVID-19, especially in culturally devout and collectivist societies, and that the intensity and adaptiveness of faith-based responses varied across contexts. Such findings underscore the need to situate coping research within specific cultural and religious frameworks.

Against this backdrop, Colombia offers a distinctive setting for examining coping strategies due to its complex sociocultural and historical landscape. As a predominantly religious society, faith plays a central role in individual and collective responses to adversity, contributing to resilience and life satisfaction (Sánchez-Fuentes et al., 2018). Moreover, empirical work in Colombia indicates that coping behaviors are deeply shaped by cultural and contextual factors (Cerquera et al., 2020), which reinforces the importance of a localized analysis of how crises are navigated. Studying coping mechanisms in this context both contributes to global debates on resilience and informs culturally sensitive mental health interventions in which religious coping is treated as a key psychosocial resource.

Accordingly, the present study examines how coping strategies, particularly religious practices, were configured and distributed among adults in Colombia in the aftermath of the COVID-19 pandemic. We first conducted a psychometric validation of a coping strategies instrument to ensure analytical rigor and then (1) identified latent coping profiles using cluster analysis on empirically validated dimensions, (2) explored how these profiles varied across sociodemographic subgroups (age, gender, socioeconomic status), and (3) assessed the role and differential integration of religious coping within these patterns through multiple-group confirmatory factor analysis.

## Methods

### Sample

This study used a non-probabilistic, volunteer sample composed of 5,559 adults residing in Santander, surveyed between June and October 2022. Inclusion criteria were being 18 years or older, living in the department, and providing informed consent. Participants were recruited in Bucaramanga, its metropolitan area, and other localities. In-person data collection was conducted by trained research staff with institutional identification who administered the questionnaires on electronic tablets after providing ethical information and obtaining consent, while online participation occurred mainly via social media, where individuals accessed study details, consent forms, and the questionnaire remotely.

To enhance heterogeneity within the constraints of convenience sampling, invitations were disseminated in diverse public and institutional settings (educational institutions, workplaces, parks) and online platforms. However, self-selection bias remains a concern, as volunteers may differ from the broader population in traits such as openness, social engagement, or typical coping styles, which may limit generalizability. Even so, the sample shows substantial variability in age, gender, marital status, and socioeconomic level (Table 1) and future research using randomized or systematic sampling is recommended to better assess the robustness of these patterns.

**Table 1:** Participant demographic characteristics (N = 5,559)

Variable	Category	M (SD) or N (%)
Age	Mean (SD)	28.11 (10.79)
	Min–Max	18–84
Gender	Male	2144 (38.57)
	Female	2566 (46.16)
	Transgender	5 (0.1%)
	Non-binary	17 (0.3%)
	Prefer not to say	827 (14.88)
Marital status	Single	3376 (60.73)
	Married	518 (9.32)
	Divorced	36 (0.6%)
	Widowed	24 (0.4%)
	Consensual union	561 (10.09)
	Prefer not to say	1044 (18.78)
Socioeconomic level	Stratum 1	591 (10.63)
	Stratum 2	1630 (29.32)
	Stratum 3	1664 (29.93)
	Stratum 4	637 (11.46)
	Stratum 5	32 (0.58)
	Stratum 6	11 (0.2)
	Prefer not to say	994 (17.88)

In accordance with the informed consent procedure, participants were allowed to withhold personal demographic information. However, all individuals included in the analysis completed the full Coping Strategies Questionnaire

### Instruments

Coping strategies were measured with a 66-item questionnaire based on Lazarus and Folkman’s (1984) model and adapted to the Colombian context by Londoño et al. (2006). Items were rated on a five-point Likert scale from “strongly disagree” to “strongly agree.” Internal consistency was examined using Cronbach’s alpha, while sampling adequacy and factorability were evaluated with the Kaiser–Meyer–Olkin (KMO) index and Bartlett’s test of sphericity. Dimensionality was examined through exploratory and confirmatory factor analyses, with validation results reported in the results section.

The final set of nine coping strategies includes eight originally proposed by Londoño et al. (2006): problem-solving (PS), passive coping (PAC), emotional avoidance (EAV), religion (REL), anger expression (ANE), seeking professional support (SPS), seeking social support (SSS), and positive reappraisal (PRE). Additionally, a new strategy, self-sufficiency (SELF), emerged as an avoidant coping mechanism, wherein individuals acknowledge their limitations but choose to deny or distract themselves from problems.

### Procedure

We applied hierarchical cluster analysis using complete linkage with squared Euclidean distance to identify subgroups of coping responses. This method constrains the maximum within-cluster dissimilarity, yielding compact, tightly bound clusters of roughly equal diameter, and reducing the chaining effect, while multiple linkage methods and distance metrics were initially explored to ensure robustness. The final solution comprised four empirically derived coping profiles.

To examine whether differences between these profiles reflected substantive psychological variation rather than measurement bias, we conducted multiple-group confirmatory factor analysis (MG-CFA) to test the structural invariance of the coping model. Classical MG-CFA based on observed covariates assumes a priori homogeneous groups and, when many small or unbalanced categories are specified, can inflate Type I error, reduce power, and obscure true heterogeneity by imposing restrictive equality constraints (Sterner & Goretzko, 2023). In contrast, data-driven subgrouping (for example, recursive-partitioning or exploratory factor-analytic trees) provides empirically grounded clusters with adequate cell sizes and a lower multiple-testing burden (Sterner et al., 2025), a logic that informed our use of clustering as the basis for MG-CFA.

Although we did not test measurement invariance across age, gender, marital status, or socioeconomic level, these variables were used to interpret the composition of each cluster, given evidence that coping, and religious coping in particular, is shaped by developmental stage (Torralba et al., 2021), gendered socialization (Ahmad & Jafree, 2023; Chatters et al., 2008), relational context (Chilon-Huaman et al., 2023), and economic precarity (Souza et al., 2024). In Latin America, faith-based coping has been shown to mediate the psychological impact of structural disadvantage, especially among socially vulnerable populations.

## Results

### Psychometric Validation of the Coping Strategies Scale

**Fig. 1:**
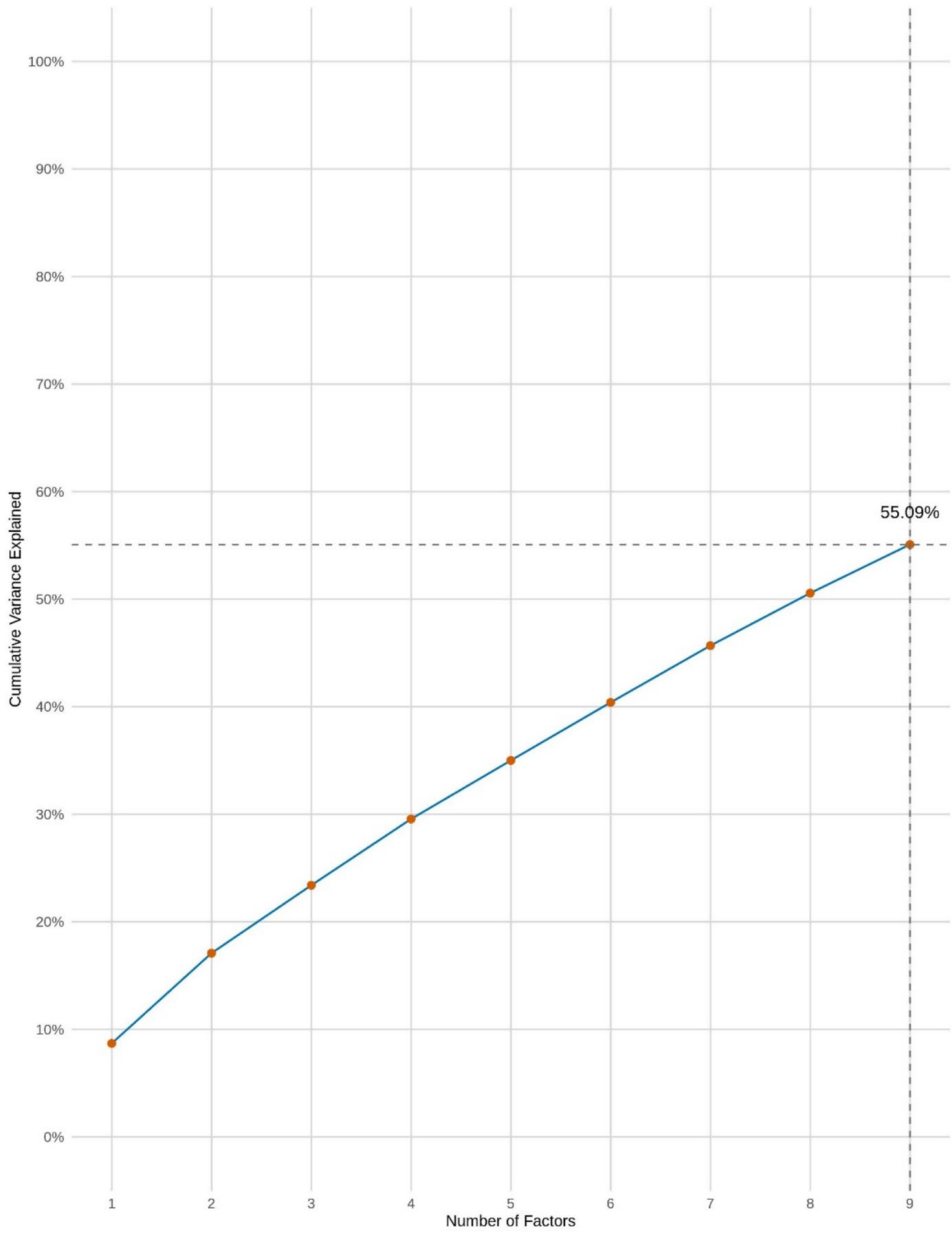
Variance versus number of factors

The initial dataset comprised 5,645 observations across 66 items and was refined to 5,559 complete cases after preprocessing to ensure consistency for factor analysis. Sampling adequacy was excellent, as indicated by an overall KMO of 0.96 and item-level values between 0.93 and 0.98, and Bartlett’s test of sphericity was significant (χ2 = 220,733.8, df = 2,145, *p* < .000), confirming the factorability of the correlation matrix. An exploratory factor analysis (maximum likelihood, Varimax rotation) using the Kaiser criterion (eigenvalues > 1) initially suggested a 12-factor solution; however, weak and cross-loadings prompted iterative refinement. A nine-factor solution was ultimately retained, balancing theoretical interpretability and statistical robustness and explaining 55.09% of the total variance, with strong communalities and well-defined factor loadings (see Fig. 1).

Oblimin rotation was then used, acknowledging that coping strategies can be related rather than strictly orthogonal. Associations between religion and other coping strategies were interpreted as contemporaneous correlations among latent constructs, not directional effects. Autonomy, originally represented by only two items, was excluded as an independent factor due to instability and was reassigned within broader coping dimensions. Confirmatory factor analysis (CFA) of the nine-factor model yielded the following fit indices: RMSEA = .062, 90% CI [.061, .062]; CFI = .816; TLI = .810; SRMR = .199. These values indicate that although RMSEA suggests acceptable approximation, CFI/TLI fall below conventional cutoffs and SRMR points to areas of misfit, implying a moderate overall fit and scope for structural refinement (for example, re-specifying residual correlations or removing poorly performing items).

To evaluate factor score reliability, regression-based multiple R^2^ coefficients were computed, ranging from 0.77 to 0.93 for the nine factors, which supports strong relationships between latent constructs and their indicators (see Table 2). Despite some limitations in global fit, the model maintains conceptual clarity and adequate statistical performance, providing a sufficiently robust measurement structure for the subsequent cluster analysis and interpretation of coping profiles.

**Table 2:** Measures of factor score adequacy

Factor	Correlation of scores with factors	Multiple R2	Minimum correlation of factor scores
1	0.96	0.93	0.86
2	0.96	0.93	0.86
3	0.95	0.89	0.79
4	0.96	0.92	0.83
5	0.96	0.92	0.84
6	0.96	0.92	0.84
7	0.95	0.9	0.8
8	0.93	0.87	0.74
9	0.88	0.77	0.55

### Cluster Analysis

Once the latent dimensions were established through the psychometric validation of the Coping Strategies Scale, the analysis focused on identifying meaningful subgroups based solely on participants’ coping responses. Hierarchical clustering was selected as the primary method due to its capacity to reveal nested structures within complex datasets (Duarte-Duarte et al., 2021). The process involved selecting an appropriate distance metric and linkage function. In this study, we employed Euclidean distance and complete linkage to generate clusters of individuals with similar coping profiles, emphasizing maximal dissimilarity between clusters while approximating similarity to a hypothetical or “average” individual within each group. The final clustering structure was established through a comprehensive combinatorial analysis that optimized both the number of groups and their internal coherence. Ultimately, group labels were informed by coping strategies in conjunction with sociodemographic characteristics, allowing for a more meaningful and theoretically grounded identification of subgroups (see Supplementary figure S1).

With homogeneous clusters established, the next step involved examining the latent relationships within each subgroup. Structural equation modeling (SEM) was conducted separately for each cluster to account for within-group measurement invariance and to identify potential structural differences. This approach acknowledged that the relationships among coping strategies may vary across subpopulations, thereby requiring tailored models to accurately reflect each group’s internal dynamics, as recommended by Rosseel (2012). By modeling each cluster independently, the analysis provided a nuanced understanding of how coping mechanisms interact within distinct psychosocial profiles.

A multiple-group confirmatory factor analysis (MG-CFA) was conducted to assess measurement invariance across the identified clusters. This procedure aimed to verify whether the factor structure, item loadings, and intercepts remained consistent across subgroups, thus ensuring the validity of cross-cluster comparisons. Following the sequential approach outlined by Byrne et al. (1989) and Chen (2008), a series of increasingly constrained models were tested: configural invariance (same factor structure), metric invariance (equal factor loadings), scalar invariance (equal intercepts), and strict invariance (equal residual variances). This stepwise testing strategy allowed for the identification of the highest level of invariance supported by the data, providing the methodological foundation for subsequent latent mean comparisons and cluster-specific structural modeling. Table 3 shows the fit indices results.

**Table 3:** Model fit indices for measurement invariance testing across clusters

Model	Df	AIC	BIC	Chisq	Chisq diff	RMSEA	Df diff	Pr(> Chisq)
Configural	6012	688,197	693,681	30,675	–	–	–	–
Weak	6156	688,877	693,407	31,643	967.5	0.0641	144	< .001***
Strong	6300	691,144	694,720	34,198	2555	0.1098	144	< .001***
Strict	6471	707,169	709,613	50,564	16,366.8	0.2611	171	< .001***

Asterisks indicate significance levels for Chi-square difference tests

^***^p < 0.001

To ensure that our nine-factor coping strategies instrument functioned equivalently across the four empirically derived clusters, we conducted a layered invariance testing procedure within the lavaan framework (v. 0.6–19, ML estimator, nlminb optimizer). First, we fitted an unconstrained configural model, allowing all parameters to vary freely in each group. This baseline model achieved adequate fit (χ^2^(6012) = 30 675.17, CFI = .844, RMSEA = .054), confirming that the same general factor structure holds across clusters and justifying further constraints. Religion showed positive correlations with several coping factors (standardized r, all *p* < .001): Seeking Professional Support 0.54; Seeking Social Support 0.38; Positive Reappraisal 0.37; Anger Expression 0.36; Problem-Solving 0.24; Denial 0.23; Emotional Avoidance 0.17. These estimates indicate co-deployment patterns rather than causal influence.

Building on this foundation, we imposed equality constraints on all factor loadings to test metric invariance. The resulting model (χ^2^(6156) = 31 642.63, CFI = .839; ΔCFI = .005) exhibited only a trivial decline in comparative fit, indicating that participants in each cluster interpret the coping dimensions similarly and that latent constructs have the same metric meaning across groups. Practically, metric invariance ensures that relationships between coping strategies and external variables (e.g., demographic predictors) can be compared without bias.

Next, to examine whether latent means are comparable, we constrained both loadings and item intercepts under the scalar invariance model. Despite an expected increase in misfit (χ^2^(6300) = 34 197.60, CFI = .824; ΔCFI = .015), the change remained within accepted thresholds (ΔCFI ≤ .02; RMSEA ≤ .06), supporting valid comparisons of average scores across clusters. In this context, establishing scalar invariance allows us to interpret mean differences.

Finally, we evaluated strict invariance by further constraining residual variances. This model (χ^2^(6471) = 50 564.37, CFI = .722; RMSEA = .070) failed to meet conventional criteria, suggesting that error variances differ modestly across clusters. While strict invariance is rarely achieved in practice, its partial rejection highlights that some items may exhibit differential measurement precision in certain subgroups.

As shown in Table 3, the establishment of both configural (χ^2^(6012) = 30,675, CFI = 0.844, RMSEA = 0.054) and weak (χ^2^(6156) = 31,643, CFI = 0.839, RMSEA = 0.054) invariance across the four identified clusters confirms that the underlying latent constructs maintain consistent structural configurations and factor loadings, supporting the assumption that these constructs are conceptually equivalent across subgroups. This implies that the measurement model functions similarly in each cluster, allowing valid comparisons of relationships between coping constructs and other variables.

Moreover, the confirmation of strong invariance (χ^2^(6300) = 34,198, CFI = 0.823, RMSEA = 0.056) indicates that latent means can be reliably compared across clusters. This level of invariance suggests that any observed differences in coping strategy levels are attributable to genuine differences in the latent constructs rather than to measurement artifacts or systematic bias.

However, the strict invariance model, which additionally constrains residual variances to equality, exhibited poor fit, with a CFI of 0.722, and RMSEA of 0.070. The sharp deterioration in fit confirms that strict invariance does not hold. This inability to establish strict invariance, which requires equivalence in residual variances across groups, suggests that measurement error varies between clusters. These discrepancies in residual variances may reflect differences in response styles, cultural interpretations of items, or contextual factors that influence the precision of item responses. While strong invariance is sufficient for latent mean comparisons between clusters, error variances are not consistent across all groups, so absolute differences analysis and its respective interpretation must be applied with caution.

Finally, our analysis supported the classification of participants into four distinct groups based on their patterns across the nine coping strategies: Cluster 1_ Emotionally Restrained Passive Coping Profile (n = 1,772), Cluster 2_ Religiously Integrated and Socially Supported Profile (n = 2,313), Cluster 3_ Highly Engaged Profile with High Spiritual Investment (n = 748), and Cluster 4_ Cognitively Oriented and Socially Withdrawn Profile (n = 726). Table 4 presents the mean scores for each coping strategy within these clusters.

**Table 4:** Average values of coping strategies for each group

Cluster	PS	PAC	EAV	REL	ANE	SPS	SELF	SSS	PRE
1	2.73	2.73	2.89	2.75	2.63	2.66	2.82	2.74	2.77
2	3.62	2.99	3.22	3.49	2.85	3.13	3.16	3.41	3.39
3	4.19	3.82	3.76	3.84	2.27	3.53	3.27	3.93	3.61
4	3.49	2.49	3.3	3.01	1.96	2.48	2.84	2.66	3.44

Problem-solving (PS); Passive Coping (PAC); Emotional Avoidance (EAV); Religion (REL); Anger Expression (ANE); Seeking Professional Support (SPS); Self-Sufficiency (SELF); Seeking Social Support (SSS); Positive Reappraisal (PRE)

Subsequent analysis of variance (ANOVA) and least significant difference tests explored intra-group differences in strategy utilization, with graphical representations (spider plots) provided for each group. This analysis revealed distinctive coping profiles, indicating varied preferences and frequencies in strategy usage across different demographic groups.

Cluster analysis identified four distinct coping profiles among adults in Santander, Colombia, with religious engagement emerging as a central component in two of these groups. The largest cluster, *Religiously Integrated and Socially Supported Profile* (Cluster 2), demonstrated high levels of religious coping alongside strong problem-solving, positive reappraisal, and social support-seeking, suggesting that spirituality was deeply integrated into a resilient and well-regulated coping style. Similarly, *Highly Engaged Profile with High Spiritual Investment* (Cluster 3) exhibited even higher reliance on religious practices, coupled with strong cognitive and emotional engagement, although their elevated levels of passive coping and emotional avoidance may signal heightened emotional strain. In contrast, *Cognitively Oriented and Socially Withdrawn Profile* (Cluster 4) relied primarily on cognitive strategies, showing moderate use of religious coping but minimal external help-seeking and emotional expressiveness. Finally, *Emotionally Restrained Passive Coping Profile* (Cluster 1) presented a more passive coping profile, with moderate emotional avoidance and limited religious involvement.

Across all clusters, gender identity was not statistically associated with group membership (χ^2^ = 20.35, df = 12, *p* = 0.0608), indicating no robust overall dependence. However, analysis of standardized residuals revealed meaningful deviations from expected distributions. Cluster 1 showed a slightly higher proportion of men than expected (standardized residual =  + 1.75) and fewer women (standardized residual =  − 1.69), while Cluster 2 exhibited the opposite trend. These nuances, while not statistically conclusive, point to latent gender-related dynamics that may influence coping orientations or identification with specific psychosocial profiles.

*Cluster 1* Emotionally Restrained Passive Coping Profile. This cluster (n = 1,772) comprises individuals with a mean age of 29.6 years. While women constituted the majority (64.5%), this group exhibited a higher-than-expected proportion of men (standardized residual =  + 1.75) relative to the overall sample distribution. Most participants were single (51.7%), though this group displayed greater marital diversity than others, and belonged primarily to mid-level socioeconomic strata (levels 3–4).

Coping strategies within this profile revealed moderately low levels of problem-solving (2.73) and positive reappraisal (2.77), combined with moderate emotional avoidance (2.89) and passive coping (2.73). Religious coping was present but at moderate levels (2.74), neither predominant nor negligible.

The unexpected overrepresentation of male participants suggests a gendered pattern of emotionally restrained coping, where men may be more inclined toward passive and internally regulated strategies. Their moderate reliance on religious coping may offer some emotional support, but the overall passive orientation suggests vulnerability to cumulative strain, particularly for men who may underutilize interpersonal and affective channels of regulation (see Table 5 and supplementary figure S2).

**Table 5:** *P* values of the ANOVA to compare coping strategies within group 1: Emotionally Restrained Passive Coping Profile

	PS	PAC	EAV	ANE	PRE	SELF	SSS	REL
PAC	0.908							
EAV	0.000*	0.000*						
ANE	0.000*	0.000*	0.000*					
PRE	0.007*	0.009*	0.000*	0.000*				
SELF	0.000*	0.000*	0.000*	0.000*	0.000*			
SSS	0.588	0.651	0.000*	0.000*	0.013*	0.000*		
REL	0.393	0.468	0.000*	0.000*	0.082	0.000*	0.677	
SPS	0.000*	0.000*	0.000*	0.006*	0.000*	0.000*	0.000*	0.000*

Problem-solving (PS); Passive Coping (PAC); Emotional Avoidance (EAV); Religion (REL); Anger Expression (ANE); Seeking Professional Support (SPS); Self-Sufficiency (SELF); Seeking Social Support (SSS); Positive Reappraisal (PRE)

^*^p value < 0.05

*Cluster 2* Religiously Integrated and Socially Supported Profile. Cluster 2 (n = 2,313) represents the largest and arguably most adaptive profile. Participants were predominantly young adults (mean age ≈ 27 years), mostly female (56.9%), largely single (60%), and primarily from mid-level socioeconomic strata (levels 3–4). This group consistently exhibited high scores across multiple coping domains, including problem-solving (3.62), positive reappraisal (3.39), seeking social support (3.41), and religious coping (3.49). Passive coping (2.99) and anger expression (2.85) remained moderate and did not dominate the profile.

While the distribution of gender identity did not differ significantly from the expected, this cluster showed a modest but meaningful overrepresentation of women (standardized residual =  + 1.45). This profile reflects a resilient and well-regulated coping orientation that actively combines cognitive, emotional, and spiritual resources. The high endorsement of religious coping highlights the instrumental role of faith not only as emotional consolation but also as an integral part of adaptive functioning. The broad coping repertoire observed in this group suggests psychological flexibility and a robust capacity for navigating adversity (see Table 6 and supplementary figure S3).

**Table 6:** P values of the ANOVA to compare coping strategies within group 2: religiously integrated and socially supported profile

	PS	PAC	EAV	ANE	PRE	SELF	SSS	REL
PAC	0.000*							
EAV	0.000*	0.000*						
ANE	0.000*	0.000*	0.000*					
PRE	0.000*	0.000*	0.000*	0.000*				
SELF	0.000*	0.000*	0.000*	0.000*	0.000*			
SSS	0.000*	0.000*	0.000*	0.000*	0.246	0.000*		
REL	0.000*	0.000*	0.000*	0.000*	0.000*	0.000*	0.000*	
SPS	0.000*	0.000*	0.000*	0.000*	0.000*	0.086	0.000*	0.000*

Problem-solving (PS); Passive Coping (PAC); Emotional Avoidance (EAV); Religion (REL); Anger Expression (ANE); Seeking Professional Support (SPS); Self-Sufficiency (SELF); Seeking Social Support (SSS); Positive Reappraisal (PRE)

^*^p value < 0.05

*Cluster 3* Highly Engaged Profile with High Spiritual Investment. This cluster (n = 748) consisted primarily of women (52.2%), with most participants in the younger to mid-adult range (mean age ≈ 28 years), predominantly single (75.8%), and largely from lower socioeconomic strata (levels 1–2). Participants in this group showed the highest scores on problem-solving (4.19) and seeking social support (3.93), as well as a strong reliance on religious coping (3.84). However, they also reported elevated levels of passive coping (3.82) and emotional avoidance (3.76), suggesting a dual pattern of engagement and internalized distress. Anger expression was moderate (2.27).

This group reflects an emotionally saturated but resourcefully engaged coping orientation. Their effective use of social, cognitive, and spiritual strategies indicates a high level of motivational investment in adaptive functioning. However, the simultaneous reliance on passive and avoidant strategies suggests possible emotional overload. Religious coping appears to serve both as a spiritual anchor and a mechanism of affective containment within this complex profile (see Table 7 and supplementary figure S4).

**Table 7:** *P* values of the ANOVA to compare the use of coping strategies within group 3: highly engaged profile with high spiritual investment

	PS	PAC	EAV	ANE	PRE	SELF	SSS	REL
PAC	0.000*							
EAV	0.000*	0.000*						
ANE	0.000*	0.000*	0.000*					
PRE	0.000*	0.000*	0.000*	0.000*				
SELF	0.000*	0.000*	0.000*	0.000*	0.000*			
SSS	0.000*	0.000*	0.000*	0.000*	0.246	0.000*		
REL	0.000*	0.000*	0.000*	0.000*	0.000*	0.000*	0.000*	
SPS	0.000*	0.000*	0.000*	0.000*	0.000*	0.086	0.000*	0.000*

Problem-solving (PS); Passive Coping (PAC); Emotional Avoidance (EAV); Religion (REL); Anger Expression (ANE); Seeking Professional Support (SPS); Self-Sufficiency (SELF); Seeking Social Support (SSS); Positive Reappraisal (PRE)

^*^p value < 0.05

Cluster 4: Cognitively Oriented and Socially Withdrawn Profile. Cluster 4 (n = 726) comprises mostly young women (56.5%), with a mean age of 27 years, predominantly single (69%), and primarily from mid-level socioeconomic strata (3–4). The defining characteristics of this profile include high levels of problem-solving (3.49) and positive reappraisal (3.44), contrasted by very low scores in anger expression (1.96) and limited use of social (2.66) and professional support (2.48). Emotional avoidance was moderate (3.30), and religious coping was lower relative to other clusters (3.01).

The distribution of gender identity was consistent with the expected values, supporting the interpretation of this cluster as a broadly distributed cognitive coping profile not specifically aligned with any gender group.

This coping pattern reflects a self-reliant, cognitively focused orientation with minimal outward emotional expression or help-seeking behavior. While a moderate engagement with religious coping suggests some access to internal spiritual resources, the limited use of interpersonal and emotional channels may pose a risk of psychological isolation (see Table 8 and supplementary figure S5).

**Table 8:** *P* values of the ANOVA to compare the use of coping strategies within group 4: cognitively oriented and socially withdrawn profile

	PS	PAC	EAV	ANE	PRE	SELF	SSS	REL
PAC	0.000*							
EAV	0.000*	0.000*						
ANE	0.000*	0.000*	0.000*					
PRE	0.204	0.000*	0.002*	0.000*				
SELF	0.000*	0.000*	0.000*	0.000*	0.000*			
SSS	0.000*	0.000*	0.000*	0.000*	0.000*	0.000*		
REL	0.000*	0.000*	0.000*	0.000*	0.000*	0.002*	0.000*	
SPS	0.000*	0.727	0.000*	0.000*	0.000*	0.000*	0.000*	0.000*

Problem-solving (PS); Passive Coping (PAC); Emotional Avoidance (EAV); Religion (REL); Anger Expression (ANE); Seeking Professional Support (SPS); Self-Sufficiency (SELF); Seeking Social Support (SSS); Positive Reappraisal (PRE)

^*^p value < 0.05

Finally, principal component analysis (PCA) was conducted to explore the latent structure of coping strategies and to support the interpretability of the clustering solution (see supplementary figure S6). The first three components accounted for 64.3% of the total variance, offering a meaningful low-dimensional representation of the underlying psychological space. The first component (38.9%) reflected a continuum from Passive Avoidance to Active Engagement, differentiating individuals who rely on withdrawal and emotional avoidance from those who engage in problem-solving, positive reappraisal, and both social and religious support-seeking. The second component (16.3%) captured variation in emotional openness, ranging from Emotional Restraint to Emotional Expression, highlighting differences in how participants externalize or suppress affect. The third component (9.1%) described an interpersonal dimension, spanning from Self-Reliance to Support-Seeking, and provided a nuanced layer that distinguishes how participants relate to others during stress.

Visualizing the clusters across these components revealed partial overlap when all groups were projected simultaneously, yet clearer patterns emerged through pairwise comparisons. For instance, Cluster 1, the Emotionally Restrained Passive Coping Profile, was concentrated in the low regions of PC1 and PC2, indicating low activation and subdued affect. Cluster 4, although also low in engagement, differed by showing stronger scores in problem-solving and reappraisal and shifted along PC3, suggesting a more self-reliant, cognitively driven style.

Meanwhile, Clusters 2 and 3 exhibited high engagement but diverged emotionally. Cluster 2, the Religiously Integrated and Socially Supported Profile, combined active coping and help-seeking with emotional regulation, while Cluster 3, the Highly Engaged Profile with High Spiritual Investment, stood out for its emotional expressiveness and elevated use of both active and passive strategies. This divergence was evident in their separation along PC2 and PC3, reflecting distinct affective and interpersonal orientations despite their shared behavioral activation.

## Discussion

This study provides empirical evidence on the heterogeneity of coping strategies in a culturally devout population (Sánchez-Fuentes et al., 2018), highlighting the central role of religious engagement in shaping distinct psychosocial profiles. These profiles capture the multidimensional nature of coping in post-pandemic Colombia, reflecting interactions between psychological strategies and sociodemographic traits.

A complementary PCA of the nine coping dimensions further illuminated the latent landscape underlying these profiles. The first three components represent Passive Avoidance to Active Engagement (38.9% variance), Emotional Restraint to Emotional Expression (16.3%), and Self-Reliance to Support-Seeking (9.1%), and accounted for 64.3% of the total variance neatly mapped onto our four clusters. This alignment between PCA axes and cluster configurations underscores that the empirically derived profiles are not only statistically robust but also grounded in meaningful psychological continua, thereby reinforcing the interpretive framework for subsequent invariance testing and comparative analyses.

Two of the four profiles showed strong integration of religious coping, underscoring the importance of spirituality as both an emotional and cognitive resource. The *Religiously Integrated and Socially Supported Profile* demonstrated high engagement with religious practices alongside robust use of problem-solving, reappraisal, and interpersonal support, which suggests a psychologically flexible and resilient configuration. These results reveal how religious practices in Colombia are interwoven with community networks and instrumental action (Kotherová et al., 2024). These findings support previous research that frames religious coping as more than an emotion-focused strategy, proposing instead a multifaceted resource capable of enhancing both emotional regulation and instrumental action (Asif et al., 2024; Kroesbergen-Kamps, 2024). The moderate use of passive coping and low anger expression within this group further suggests that faith may contribute to emotional stability and impulse regulation.

These results align with a recent meta-analysis by Pankowski and Wytrychiewicz-Pankowska (2023), which found that religious coping increased globally during the COVID-19 pandemic and was particularly prominent in culturally devout and collectivist societies. Their analysis showed that positive religious coping was consistently associated with improved psychological outcomes, including reduced stress and greater well-being. Our findings contribute to this literature by demonstrating how religious engagement in Colombia formed part of differentiated coping configurations that varied in their psychological and social adaptiveness.

The *Highly Engaged Profile with High Spiritual Investment* displayed an even stronger religious reliance, paired with elevated use of social and cognitive strategies. However, this profile also exhibited the highest levels of emotional avoidance and passive coping, suggesting an emotionally saturated configuration. Religious coping in this context may function as a double-edged resource: offering support but also signaling high emotional demand. This dual pattern mirrors previous findings suggesting that religious engagement can be mobilized under high distress to maintain perceived control and coherence (Waila & Lindsay, 2024).

By contrast, the *Emotionally Restrained Passive Coping Profile* was marked by lower use of most coping strategies, including limited engagement with religious practices. Despite a female majority in absolute terms, this cluster had a higher-than-expected proportion of men, suggesting a possible gendered pattern of emotional containment and low help-seeking. Men in this group may rely on stoic or self-contained strategies that eschew overt emotional expression or spiritual recourse. This aligns with prior research documenting male tendencies toward avoidance and underutilization of affective resources, especially in culturally normative contexts where masculine identity may discourage emotional vulnerability (Chew et al., 2020; Kéri, 2024).

The *Cognitively Oriented and Socially Withdrawn Profile* reflected a highly instrumental but interpersonally detached approach, with strong use of problem-solving and reappraisal but limited engagement with social or religious support. The limited use of religious coping in this group reinforces the idea that although faith is culturally salient, it is not universally adopted across all subpopulations (Hvidtjørn et al., 2014).

Gender emerged as a key demographic variable influencing coping orientation. While overall gender identity was not statistically associated with group membership, residual analyses revealed meaningful patterns. Women were overrepresented in the two spiritually engaged profiles, consistent with broader cross-cultural trends linking female gender with higher religious involvement and emotional engagement (Ahmad & Jafree, 2023; Chatters et al., 2008; Hvidtjørn et al., 2014). Conversely, men were more prevalent in emotionally restrained or self-sufficient profiles, which align with literature on masculine coping norms that emphasize independence and emotional control (Hamid et al., 2023). These patterns highlight the need for gender-sensitive mental health approaches that validate women’s use of spiritual resources while encouraging men to adopt more diversified and emotionally expressive coping strategies.

From a practical standpoint, these findings offer important implications for culturally sensitive mental health interventions in Colombia, a context characterized by high religiosity and socioeconomic vulnerability (Sánchez-Fuentes et al., 2018). Integrating religious coping into therapeutic frameworks through faith-informed counseling, spiritually grounded cognitive reframing, or community-based pastoral support, may enhance the cultural congruence and psychological effectiveness of interventions for individuals aligned with spiritually engaged profiles (Asif et al., 2024; Kroesbergen-Kamps, 2024). At the same time, interventions targeting individuals who are emotionally withdrawn or cognitively self-sufficient should incorporate affective and interpersonal dimensions, fostering greater openness to emotional expression and external support systems (Chew et al., 2020). The observed gendered patterns of coping suggest that such efforts must also be sensitive to prevailing norms of emotional restraint, particularly among men. Nevertheless, caution is warranted: These profiles should not be used prescriptively in clinical practice until further validation is achieved. Replication studies, ideally employing longitudinal or experimental designs, are necessary to assess the temporal stability and modifiability of these coping configurations.

Overall, our findings support the view that religious coping is not a monolithic or exclusively emotion-focused strategy, but rather an adaptive, contextually embedded resource that operates in concert with cognitive, behavioral, and demographic factors (Waila & Lindsay, 2024). In high-stress environments marked by economic and emotional precarity, such as those intensified by the COVID-19 pandemic, spiritual engagement may serve to complement or compensate for deficits in other domains of coping. Future research should continue to examine how faith functions within integrated repertoires of adaptation, particularly in culturally devout societies where religion not only shapes individual beliefs but also reinforces collective resilience through shared meaning and social cohesion (Kotherová et al., 2024; Pankowski & Wytrychiewicz-Pankowska, 2023; Tausch et al., 2011).

## Limitations and Future Directions

This study offers important insights into the role of religious coping in a Colombian context; however, several limitations warrant consideration. Its non-probabilistic sampling within a single Colombian region may constrain the generalizability of findings to other regions with different religious traditions, levels of rurality, or access to psychosocial resources. Additionally, cross-sectional design prevents conclusions about the temporal stability of coping profiles.

Future research should include more diverse populations across regions, adopt longitudinal designs to assess the evolution of coping dynamics over time, and incorporate qualitative or mixed-method approaches to capture the richness of religious and spiritual experiences. We believe that such efforts will enhance the cultural and psychological validity of faith-informed interventions for addressing crises.

## Supplementary Information

Below are the links to the electronic supplementary material.

Supplementary file1 (JPG 3821 KB)

Supplementary file2 (JPG 138 KB)

Supplementary file3 (JPG 137 KB)

Supplementary file4 (JPG 139 KB)

Supplementary file5 (JPG 136 KB)

Supplementary file6 (JPG 1082 KB)

## Data Availability

Data are available through Universidad Cooperativa de Colombia’s repository at: 10.57924/BNTLCN.
